# Evolution and dynamics of megaplasmids with genome sizes larger than 100 kb in the *Bacillus cereus* group

**DOI:** 10.1186/1471-2148-13-262

**Published:** 2013-12-02

**Authors:** Jinshui Zheng, Donghai Peng, Lifang Ruan, Ming Sun

**Affiliations:** 1 State Key Laboratory of Agricultural Microbiology, College of Life Science and Technology, Huazhong Agricultural University, Wuhan 430070, China

**Keywords:** Megaplasmid, Minireplicon, TubZ/TubR, pXO1-14/pXO1-16, *Bacillus cereus* group

## Abstract

**Background:**

Plasmids play a crucial role in the evolution of bacterial genomes by mediating horizontal gene transfer. However, the origin and evolution of most plasmids remains unclear, especially for megaplasmids. Strains of the *Bacillus cereus* group contain up to 13 plasmids with genome sizes ranging from 2 kb to 600 kb, and thus can be used to study plasmid dynamics and evolution.

**Results:**

This work studied the origin and evolution of 31 *B. cereus* group megaplasmids (>100 kb) focusing on the most conserved regions on plasmids, minireplicons. Sixty-five putative minireplicons were identified and classified to six types on the basis of proteins that are essential for replication. Twenty-nine of the 31 megaplasmids contained two or more minireplicons. Phylogenetic analysis of the protein sequences showed that different minireplicons on the same megaplasmid have different evolutionary histories. Therefore, we speculated that these megaplasmids are the results of fusion of smaller plasmids. All plasmids of a bacterial strain must be compatible. In megaplasmids of the *B. cereus* group, individual minireplicons of different megaplasmids in the same strain belong to different types or subtypes. Thus, the subtypes of each minireplicon they contain may determine the incompatibilities of megaplasmids. A broader analysis of all 1285 bacterial plasmids with putative known minireplicons whose complete genome sequences were available from GenBank revealed that 34% (443 plasmids) of the plasmids have two or more minireplicons. This indicates that plasmid fusion events are general among bacterial plasmids.

**Conclusions:**

Megaplasmids of *B. cereus* group are fusion of smaller plasmids, and the fusion of plasmids likely occurs frequently in the *B. cereus* group and in other bacterial taxa. Plasmid fusion may be one of the major mechanisms for formation of novel megaplasmids in the evolution of bacteria.

## Background

Horizontal gene transfer (HGT) is the major driving force of bacterial evolution [1]. Plasmids play important roles in this process via their conjugative capability [2]. Additionally, plasmids harbor genes involved in niche specific processes, and are important for bacterial adaptation to changing environmental conditions [3,4]. As plasmids can transfer frequently among different bacterial strains, they display strain-dependent distributions. Some bacterial strains containing no plasmids, while others have many; sometimes more than 20 [5]. Moreover, the same host can harbor plasmids with a wide size range. For example, *B. thuringiensis* strain CT-43 has 10 plasmids ranging from 6 kb to 300 kb [6]. However, the origin and evolution of these plasmids remains unclear. To date, studies on the evolution and dynamics have mainly focused on plasmids that have broad host ranges and harbor antibiotic-resistance (AR) genes, for example the plasmids of the incompatibility groups IncW [7], IncU [8], IncP [9,10] and PromA [11]. These plasmids usually have small genome sizes and few of them are larger than 100 kb. Information on the evolution and dynamics of plasmids that have relatively narrow host range is scarce, especially for megaplasmids larger than 100 kb. It was therefore the aim of this study to elucidate the origin, evolution and dynamics of megaplasmids with relatively narrow host range using the *Bacillus cereus* group as a model.

The *B. cereus* group includes *B. anthracis*, the causative agent of anthrax and a potential biological weapon; *B. cereus*, a ubiquitous soil bacterium and foodborne pathogen; *B. thuringiensis*, which produces insecticidal crystal proteins; and four additional species, *B. cytotoxicus*, *B. mycoides*, *B. pseudomycoides*, and *B. weihenstephanensis*[12,13]. Strains of this group typically contain several plasmids, with some strains containing more than 10 [6,14,15]. Plasmids of this group are crucial for the phenotype and virotype of strains. *B. anthracis, B. cereus*, and *B. thuringiensis* were defined mainly on the basis of plasmid-encoded phenotypic features [16-18]. Usually, these plasmids are larger than 100 kb. Indeed, most strains of this group contain one or more megaplasmids larger than 100 kb. Only two of these megaplasmids have been studied in depth. One is pXO1 (182 kb) from *B. anthracis* which harbors two minireplicons that support replication of the plasmid: *repX*[19] and *pXO1-14/pXO1-16*[20]. A minireplicon represents the smallest replication region that supports plasmid replication, and contains the origin of replication and genes encoding replication proteins. The origin of replication of plasmid is a particular sequence in a plasmid genome at which replication is initiated. The other well-studied megaplasmid is *B. thuringiensis* plasmid pBtoxis (128 kb), whose minireplicon consists of two genes: *orf156* and *orf157*[21]. The availability of more than 30 sequences of megaplasmids in genomes of the *B. cereus* group allows the investigation of their evolution and dynamics.

We collected the genome sequences of plasmids for bioinformatic analyses. First, we studied the distribution of minireplicons for all the megaplasmids. Second, we studied the relationships among different megaplasmids from the same host strain, and from all strains of the *B. cereus* group. We also studied the distributions of known minireplicons among all plasmids outside of the *B. cereus* group whose genome sequences were available.

## Results and discussion

### Six types of minireplicon exist in the megaplasmids of the *B. cereus* group

The minireplicons are the core part of plasmids and drive plasmid replication and propagation. Their diversity and evolution directly reflects the dynamics and evolution of plasmids [7,10,22,23]. Strains in the *B. cereus* group are rich in plasmid content, with plasmid numbers ranging from zero to 13 and sizes ranging from 2 kb to 600 kb [6,15,24]. Thus, the *B. cereus* group is an ideal model to study plasmid dynamics and evolution. This study aimed to characterize the origin, evolution and dynamics of megaplasmids with genome sizes larger than 100 kb by studying the distribution and evolution of their minireplicons.

We collected sequences of 56 plasmids with genome sizes ranging from 20 kb to 600 kb (Additional file 1: Table S1), including 31 megaplasmids larger than 100 kb. Among these megaplasmids, 65 putative minireplicons were identified and could be classified into six types (Table 1). Two of the six types contain two replication essential protein coding genes. One type of minireplicon, which was first reported to support the replication of *B. thuringiensis* plasmid pBtoxis [21], *tubZ/tubR*, encodes TubZ/TubR proteins, which are FtsZ-like (TubZ) and DNA-binding proteins (TubR), respectively. The second type of minireplicon, which was first reported to support the replication of *B. anthracis* plasmid pXO1, encodes essential proteins belonging to the replication initiator protein (pXO1-16) and DNA-binding protein (pXO1-14) groups, respectively [20,21]. The other four types (*ori44*, *ori60*, *rep26* and *repA_N*) encode four different essential single replication proteins, respectively [23,25]. Among these six types of minireplicon, *tubZ/tubR*, *pXO1-14/pXO1-16* and *rep26* only exist in megaplasmids, whereas the other three occur in both megaplasmids and plasmids smaller than 100 kb.

**Table 1:** Type and number of minireplicons on plasmids larger than 20 kb

**Plasmid**	**Number of minireplicon**	**Minireplicon type**	**Plasmid size (bp)**
pAH1134_566	3	*rep466*, *pXO1-16/pXO1-14*(2)	565,964
pE33L466	3	*rep466*, *rep26*, *pXO1-16/pXO1-14*	466,370
pBMB431	2	*rep466*^b^, *pXO1-16/pXO1-14*	431,971
pBWB401	5	*ori44*, *repA_N*, *rep466*, *pXO1-14/pXO1-16*(2)	417,054
pBMB400	2	*rep466*, *pXO1-16/pXO1-14*	416,210
pBMB171	3	*rep466*, *rep26*, *pXO1-16/pXO1-14*(2)	312,963
pBMB302	2	*orf156/orf157*, *pXO1-16/pXO1-14*	302,255
pBMB293	2	*orf156/orf157*, *pXO1-16/pXO1-14*	293,574
p03BB108_282	1	*rep228*^a^	282,009
pCT281	2	*orf156/orf157*, *pXO1-16/pXO1-14*	281,231
pAH820_272	2	*repX*, *pXO1-16/pXO1-14*	272,145
pPER272	2	*repX*, *pXO1-16/pXO1-14*	272,145
pAH187_270	2	*repX*, *pXO1-16/pXO1-14*	270,082
pH308197_258	2	*repX*, *pXO1-16/pXO1-14*	258,484
pBc239	2	*repX*, *pXO1-16/pXO1-14*	239,246
p03BB108_239	2	*rep466*, *pXO1-16/pXO1-14*	238,933
pBMB228	2	*rep228*, *pXO1-16/pXO1-14*	228,003
pG9842_209	2	*rep228*, *pXO1-16/pXO1-14*	209,488
pBC210	2	*rep466*, *rep26*, *pXO1-16/pXO1-14*	209,385
pBc10987	2	*repX*, *pXO1-16/pXO1-14*	208,369
pBCXO1	2	*repX*, *pXO1-16/pXO1-14*	190,861
pBMB26	2	*rep26*^**c**^, *pXO1-16/pXO1-14*	187,880
pCI-XO1	2	*repX*, *pXO1-16/pXO1-14*	181,907
pXO1	2	*repX*, *pXO1-16/pXO1-14*	181,677
p03BB102_179	2	*repX*, *pXO1-16/pXO1-14*	179,680
pBMB28	2	*ori44*, *rep466*	139,013
pG9842_140	-	NA	140,001
pBMB137	2	*ori44*, *ori60*	137,573
pBtoxis	2	*orf156/orf157*, *pXO1-16/pXO1-14*	127,923
pCT127	2	*ori60*, *repA_N*	127,885
pBMB95	1	*ori60*	95,983
pXO2	1	*repA*	94,829
pCI-XO2	1	*repA*	94,469
p03BB108_86	1	*repA*	85,879
pCT83	1	*ori44*	83,590
pBT9727	1	*repA*	77,112
pBWB402	1	*repA*	75,107
pBMB74	1	*repA*	74,480
pH308197_73	1	*ori60*	72,792
pCT72	1	*ori43*	72,074
pAW63	1	*repA*	71,777
pBMB67	1	*ori43*	67,159
pBMB65	1	*ori44*	65,873
pBWB403	-	NA	64,977
pBMB64	1	*ori43*	64,522
pLVP1401	-	NA	56,149
pALH1	-	NA	55,939
pFR55	1	*ori44*	55,712
pE33L54	-	NA	53,501
pBWB404	-	NA	52,830
pBc53	1	*ori60*	52,766
pCT51	-	NA	51,488
pBMB46	-	NA	46,634
pAH187_45	1	*ori44*	45,173
p03BB108_42	1	*ori60*	42,470
pH308197_29	-	NA	29,189

^a^ and ^b^: The two new types of TubZ/TubR minireplicon identified in this study (supporting methods and results).

^c^: Unpublished, validated by our group.

(2): Number of minireplicons of the same type on the same plasmid.

NA: no reported minireplicon.

**Figure 1:**
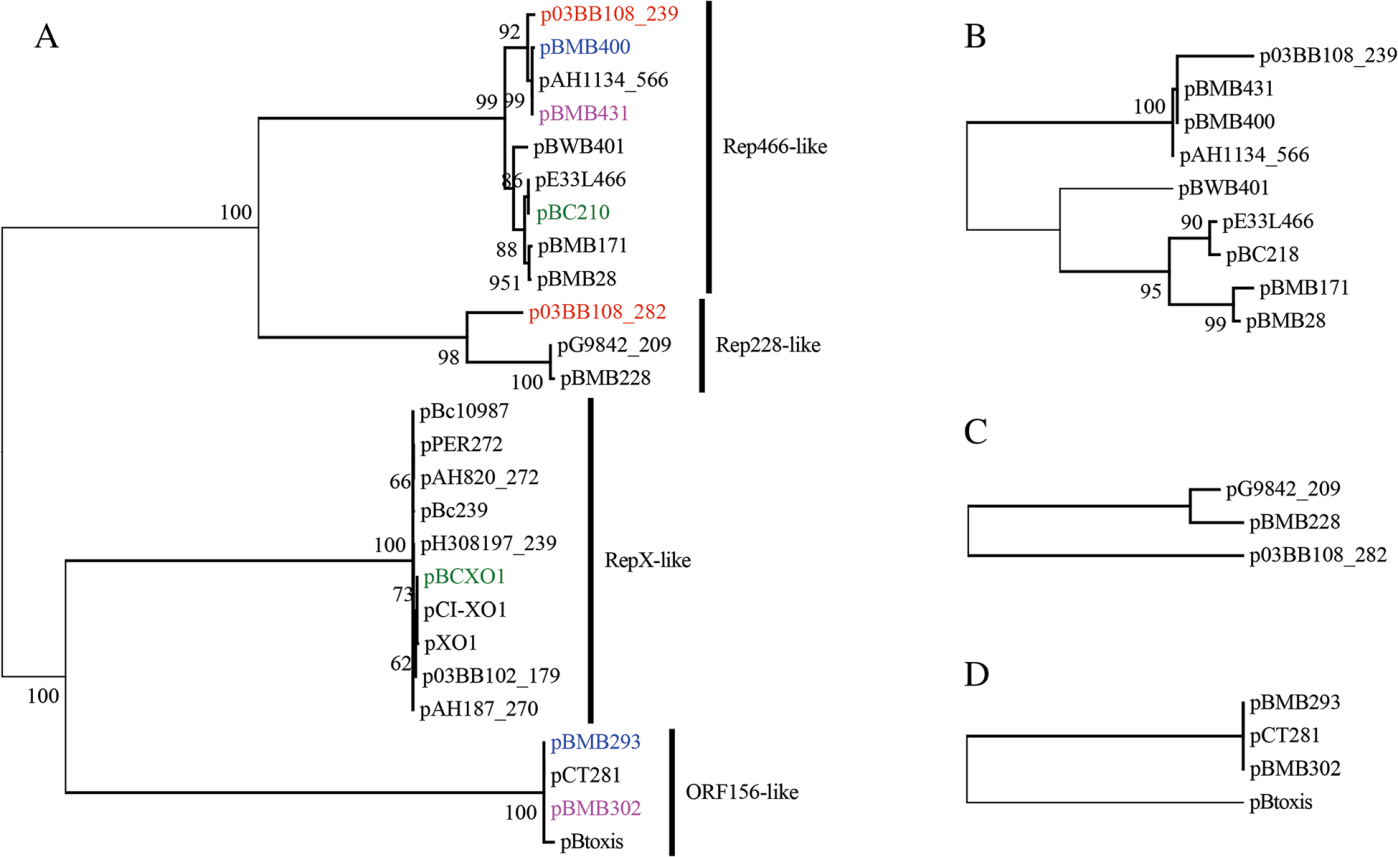
**Phylogenetic trees constructed using the ML method based on TubZ (A) protein sequences and *tubR *DNA sequences (B for *rep466*-like, C for *rep228*-like and D for *orf156/orf157*-like) from plasmids of the *B. cereus *group species.** The four subtrees in **(A)** represent the four subtypes of TubZ/TubR minireplicons. Plasmids from the same strain are marked in the same color. The number at each branch point represents the percentage of bootstrap support calculated from 1,000 replicates, and only those values higher than 50 are shown.

The minireplicon *tubZ/tubR* is distributed widely among the megaplasmids and is found in 26 of the 31 megaplasmids (Table 1). A phylogenetic tree was constructed based on the 26 TubZ protein sequences (Figure 1A). Four different clades were formed and were supported by high bootstrap values (100%). Coincidentally, four of the TubZ proteins for which a function in replication was validated, RepX in plasmid pXO1 [19], ORF156 in plasmid pBtoxis [21], Rep228-TubZ in plasmid pBMB228 and Rep466-TubZ in plasmid pBMB28 are located in the four different clades, respectively. The replication function of Rep228-TubZ and Rep446-TubZ were validated in this study (see Additional file 2). We divided all 26 *tubZ/tubR* minireplicons into four subtypes: *repX*-like, *orf156*/*orf157*-like, *rep228*-like and *rep466*-like. Among the four subtypes, only minireplicon *repX*-like encodes an orphan TubZ protein, while the other three encode not only TubZ proteins, but also TubR proteins. TubR proteins from different subtypes show no similarity to each other. However, when the gene sequences of TubR within each subtype were inspected, we found that the topologies of the phylogenetic trees showed similarities to those of the corresponding TubZ trees (Figures 1B, C and D), respectively. The DNA sequences of the origins of replication are rich in A + T and usually contain direct or invert repeats were additionally examined. The four minireplicon subtypes of *tubZ/tubR* have four different secondary structures of them, with different direct or inverted repeats (Additional file 3: Figure S4). We therefore suggest that for each subtype of *tubZ/tubR* minireplicon, their TubZ, TubR proteins and the corresponding origin of replication underwent a concerted evolution.

**Figure 2:**
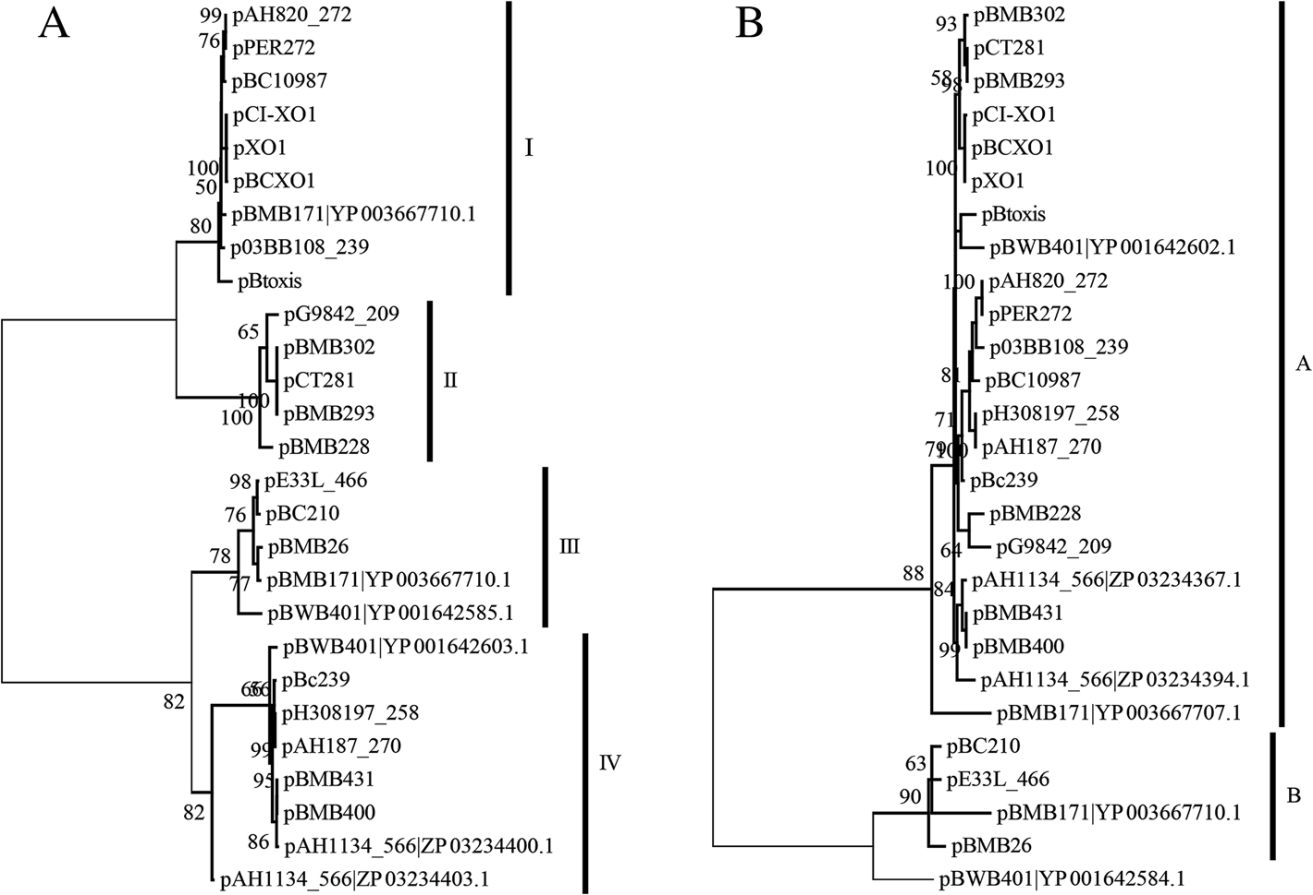
**Phylogenetic trees constructed using the ML method based on pXO1-14 (A) and pXO1-16 (B) -like protein sequences from *pXO1-14/pXO1-16*-like minireplicons from *B. cereus *group species.** When there are two or more similar sequences on the same plasmid, their accession numbers are to the right of the plasmid name. Numbers at each branch point represent the percentage of bootstrap support calculated from 1000 replicates, and only those values greater than 50 are shown.

The minireplicon *pXO1-14/pXO1-16* was found in 24 megaplasmids and three of these megaplasmids harbor two copies. When comparing the pXO1-14-like and pXO1-16-like protein sequences encoded by those 27 minireplicons, the sequence identities among pXO1-14-like proteins ranged from 40% to 100%. However, the pXO1-16-like proteins showed significantly greater conservation (P < 2.2e^-16^, Mann–Whitney test), with sequence identities>65%. When phylogenetic trees based on these two families of protein sequences were constructed (Figure 2), the topologies of the two trees were incongruent, except for some in-group topologies. For the pXO1-14-like tree, four major subgroups were supported by high bootstrap values (Figure 2A). However, two subgroups were identified for pXO1-16 (Figure 2B). This indicates that genes encoding the pXO1-14-like and pXO1-16-like proteins evolved independently and multiple recombination events have occurred in this minireplicon.

The other four minireplicons contain one essential protein each. Minireplicon *rep26* was found in four megaplasmids larger than 100 kb. In contrast, *ori44*, *ori60* and *repA_N* were found in both megaplasmids and plasmids smaller than 100 kb and are more widely distributed in smaller plasmids. For example, minireplicon *ori44* occurs in eight plasmids, only two of which are larger than 100 kb, while minireplicon *ori60* is contained by six plasmids, only one of which is larger than 100 kb. Although the two plasmids containing *repA_N* in this study are larger than 100 kb, plasmids from other Gram-positive bacteria that contain this type of minireplicon are usually smaller than 100 kb [23].

### Megaplasmids larger than 100 kb contain two or more minireplicons in *B. cereus* group

**Figure 3:**
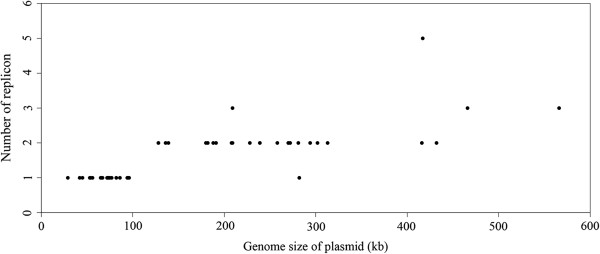
**Plot of minireplicon number against plasmid size.** Plasmids with a genome size larger than 100 kb contain two or more minireplicons.

Twenty-nine of the 31 megaplasmids larger than 100 kb contain two types of minireplicons (Table 1). Among them, 26 contain *pXO1-14/pXO1-16*-like minireplicons and one subtype of minireplicon *tubZ/tubR*. The other three have different combinations of minireplicons. The 127 kb plasmid pCT127 contains minireplicon *ori60* and *repA_N*, while the 139 kb plasmid pBMB28 contains *ori44* and *rep466*, and the 137 kb plasmid pBMB137 contains *ori44* and *ori60*. However, there are only two exceptions, pG9842_140 (140 kb) and p03BB108_282 (282 kb). No validated minireplicon was identified in plasmid pG9842_140, which indicates that it may contain novel minireplicon(s). The sequence of plasmid p03BB108_282 is incomplete. It remains thus unclear whether the single identified minireplicon *rep228* supports replication of this plasmid or whether it contains an unidentified (novel) minireplicon. Indeed, most plasmids larger than 100 kb harbor two or more minireplicons (Figure 3), whereas plasmids smaller than 100 kb usually harbor only one. Moreover, three of five megaplasmids larger than 400 kb have three or more minireplicons. In the 417 kb plasmid pBWB401; there are five coexisting minireplicons of four different types.

In plasmids with more than one minireplicon, it is not known whether all the minireplicons are functional for plasmid replication, partitioning and maintenance. For plasmid pXO1, early studies confirmed that two different types of minireplicons, *pXO1-14/pXO1-16*[20] and *repX*[19], are functional for its replication. Recently, both the *repX* and *pXO1-14/pXO1-16* minireplicons were proven to independently support replication of the full-length pXO1 plasmid, with *pXO1-14/pXO1-16* being more effective than *repX*[26]. Moreover, a 4848-bp DNA fragment within minireplicon *pXO1-14/pXO1-16* can be used to deprive plasmid pXO1 from *B. anthracis* using plasmid incompatibility [27]. This suggests that minireplicon *pXO1-14/pXO1-16* is predominantly used for plasmid replication. We therefore speculate that when there is more than one minireplicon on the same plasmid, some of them are more relevant than others. However, how these minireplicons cooperate with each other is not clear.

Minireplicons are conserved during the evolutionary history of a plasmid; however, it would be interesting to determine the evolutionary relationship of multiple minireplicons on the same plasmid. To investigate this, we conducted a comparative analysis between minireplicons *tubZ/tubR* and *pXO1-14/pXO1-16*. First, we considered the relative position of the two minireplicons on the same plasmid. When there are only two types of minireplicon on one plasmid, the distance between the minireplicons ranged from 20 to 40 kb, and the distance between two minireplicons on larger plasmids is not larger (Spearman’s r = 0.17, P = 0.41). Multiple minireplicons are frequently clustered in a certain region of the plasmid, which can be recognized as the core region for replication and maintenance. Second, we compared the three phylogenetic trees that were constructed based on protein sequences of TubZ, pXO1-14-like and pXO1-16-like (Figures 1A and 2). The major topologies of these trees were inconsistent. The pXO1-14 or pXO1-16 trees cannot support the classification of four subtypes of TubZ. Plasmids with the same subtype of *tubZ/tubR* were usually found to have different subtypes of *pXO1-14/pXO1-16*. This indicates that different minireplicons on the same plasmid evolved independently.

### Megaplasmids may be formed by fusion of smaller plasmids in *B. cereus* group

**Figure 4:**
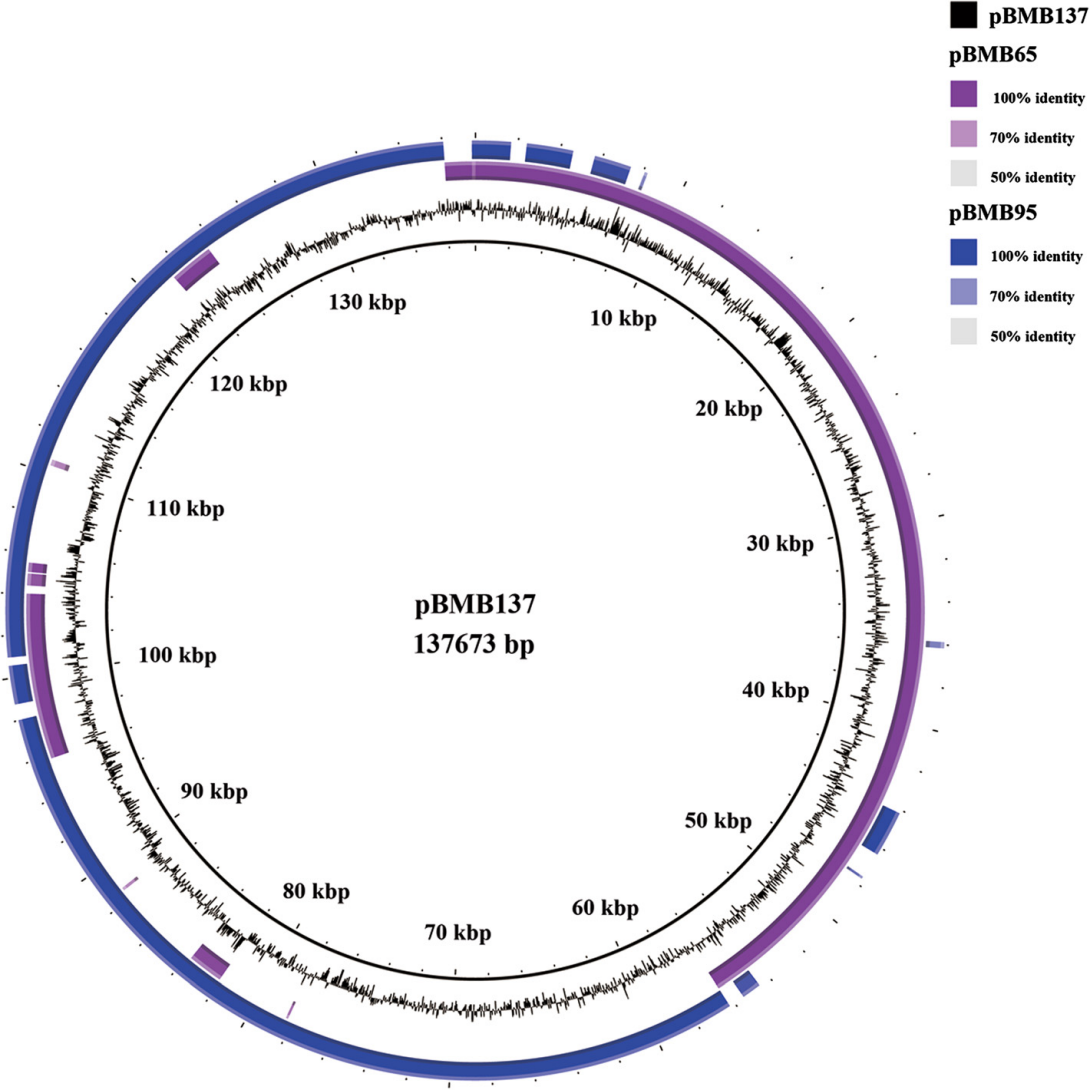
**Comparison of the genomes of pBMB137, pBMB65 and pBMB95.** From the inside: pBMB137, pBMB65 and pBMB95.

As mentioned above, two or more different putative minireplicons generally occur in the same megaplasmids in *B. cereus* group. This may indicate that these megaplasmids have resulted from the integration of two or more smaller plasmids. Minireplicons of the four *tubZ/tubR* subtypes and *pXO1-14/pXO1-16* were not found in plasmids with only one minireplicon. Of the megaplasmids whose genome sequences were available, we observed that minireplicon *pXO1-14/pXO1-16* frequently coexists with one of the four *tubZ/tubR* subtypes. These megaplasmids may share similar origins and are probably the result of a fusion between an ancestral *pXO1-14/pXO1-16*-like plasmid and an ancestral *tubZ/tubR* plasmid early in evolutionary history. For other megaplasmids, such as those containing *ori44*, *ori60* and *repA_N*, the minireplicons they contained were also found on smaller plasmids which usually have only one minireplicon. These minireplicons thus exist as sole replicon for small plasmids and as one of several minireplicons on megaplasmids. Direct evidence for this situation is provided by comparing pBMB137 of *B. thuringiensis* YBT-1520 to pBMB65 and pBMB95 of *B. thuringiensis* HD1. Plasmid pBMB137 has a genome size of 137,573 bp and contains the minireplicons *ori44* and *ori60. B. thuringiensis* HD1 harbours the 65 kb plasmid pBMB65 with minireplicon *ori44*, and the 95 kb plasmid pBMB95, with minireplicon *ori60*. The genome sequence of pBMB137 can be divided into two fragments, one of which is virtually identical to pBMB65, and the other shows a high level of similarity to pBMB95 (Figure 4). Unlike the ancestral event that formed the pXO1-like plasmids, this fusion is a recent event as the separate and smaller plasmids are maintained by some strains while others maintain with the integrated megaplasmid.

Analysis of the relationships between minireplicon types and plasmid sizes revealed that plasmids with one minireplicon are usually smaller than 100 kb. However, when two or more minireplicons were present on the same plasmid, the genome size could exceed that of either of the presumed original plasmids, usually larger than 100 kb (Table 1). For example, plasmids containing minireplicon *ori44* only have genome sizes from 45 to 85 kb. In contrast, other plasmids that combine *ori44* and one or more additional minireplicons usually have a genome size larger than 100 kb, even up to 417 kb for the plasmid pBWB401. This indicates that by integrating different minireplicons into a single plasmid, the new plasmid is capable of carrying more genes.

Larger plasmids have lower copy numbers than smaller ones [15]. Formation of larger plasmids by fusion of smaller plasmids thus reduces the amount of DNA that is required for similar plasmid genome sizes. This could provide an evolutionary advantage by reducing the energy requirement for plasmid synthesis and maintenance. Moreover, plasmids with some minireplicons have very low copy numbers, and additional minireplicons are needed to support them to replicate effectively. For example, plasmid pXO1 with only the *repX* minireplicon was reported to have copy numbers ranging from 0.8 to 1. This indicates that this minireplicon cannot effectively support plasmid replication [26]. If the plasmid contains another minireplicon, *pXO1-14/pXO-16,* in addition to the *repX* minireplicon, copy numbers ranging from 3 to 3.6 were observed and the plasmid is stably inherited. For those minireplicons that support effective plasmid replication, there may be dynamic equilibrium between the existence of small plasmids with individual minireplicons and the integration into megaplasmids with multiple minireplicons on plasmids. The selective forces driving plasmid evolution includes factors that determine the fitness of plasmids evolving as autonomous genetic elements as well factors that determine the added ecological fitness of the bacterial host. Ecological determinants that shape maintenance of small plasmids with one replicon or the integration into megaplasmids with multiple replicons are not clear.

### Compatibility groups of megaplasmids may depend on each of their minireplicons at the subtype level

Megaplasmids contain more than one minireplicon and many strains contain more than one such megaplasmid, therefore, several minireplicons co-exist in the same host. To determine the compatibility of different minireplicons, we investigated the patterns of coexistence of minireplicons *tubZ/tubR* and *pXO1-14/pXO1-16*. As shown in Figure 1 and Additional file 3: Figure S4, each *tubZ/tubR* from the same strain belongs to one of the four subtypes, with different *tubZ*s, *tubR*s and putative origins of replication. For example, the two *tubZ/tubR*s on the two megaplasmids pBC210 and pBCXO1 of *B. cereus* G9241 belong to the *repX* and *rep228* subtypes, respectively. Many strains have more than one *pXO1-14/pXO1-16* minireplicon. In most cases, each of their encoded pXO1-14 or pXO1-16 proteins from a certain strain was found to belong to different subgroups. For example, pXO1-14-like proteins encoded by plasmids pBC210 and pBCXO1 in *B. cereus* G9241 belong to subgroups I and III, respectively (Figure 2A). Their two corresponding pXO1-16-like proteins are also allocated to the two different subgroups, as shown by the pXO1-16-like protein tree (Figure 2B). In other instances, different plasmids in the same host contain pXO1-14-like or pXO-16-like proteins but only one of the two belongs to the same subgroup. For example, the two pXO1-16-like proteins of pBMB293 and pBMB400 from *B. thuringiensis* YBT-1520 are located on different branches of the same subgroup (Figure 2B), while the corresponding pXO1-14-like proteins show greater diversity and were allocated into subgroups II and IV, respectively. There was only one instance where different pXO1-14 and pXO1-16-like proteins from different minireplicons in the same strain were grouped together. Plasmid pAH1134_566 contains two pXO-14/16-like minireplicons and both proteins belong to the same subgroup. This may result from gene duplication and indicates that minireplicons of the same subgroups are compatible if they are located on the same plasmid.

All plasmids of a bacterial strain must be compatible. The minireplicon *tubZ/tubR* has four subtypes; thus, there may be four natural incompatibility groups for *tubZ/tubR*-containing megaplasmids in the *B. cereus* group. Different groups have different TubZs, TubRs and putative origins of replication. For *pXO1-14/pXO1-16*-like minireplicons, as the two essential proteins they encode do not have a concerted evolution, the putative incompatibility groups appear to be determined by the subgroup types of pXO1-14 or/and pXO1-16. Many plasmids contain both of these minireplicons; however, details regarding the coexistence of these plasmids are not clear.

### Integrated events are general among plasmids during their evolutionary histories

Plasmids outside of the *B. cereus* group with more than one minireplicon have been reported, and the most frequently mentioned were plasmids belonging to incompatibility group F (IncF). Most plasmids of this group harbor two or more minireplicons, suggesting that plasmids fusion events occurred in the evolutionary histories of these plasmids [28]. The direct example is plasmid pIP1206, which may have resulted from recombination between pRSB107 and a pAPEC-O1-ColBM-like plasmid. Among its 151 open reading frames, 56 (37%) were also present in pRSB107 and 44 (29%) in pAPEC-O1-ColBM (24) [29].

**Figure 5:**
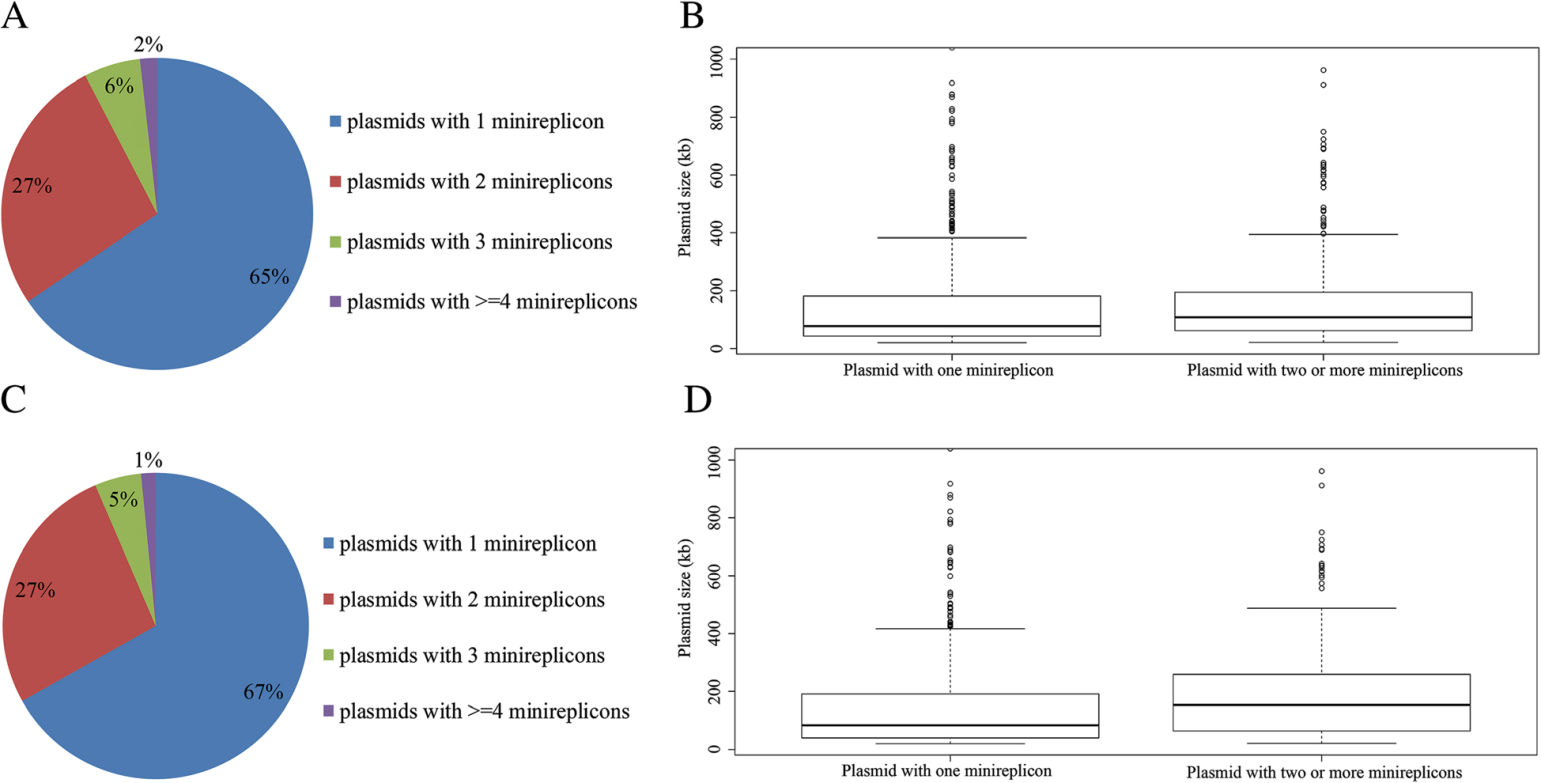
**Integrated events are general among plasmids during their evolutionary histories. (A)** One third of all the plasmids analyzed contain two or more minireplicons, **(B)** Plasmids with two or more minireplicons are larger than those with only one (P = 1.4e^-7^, Mann–Whitney test), **(C)** One third of the selected plasmids analyzed contain two or more minireplicons, **(D)** Plasmids in the selected dataset with two or more minireplicons are larger than those with only one (P = 1.035e^-06^, Mann–Whitney test).

In addition to analyzing plasmids of the *B. cereus* group, we analyzed the putative fusion events among all bacterial plasmids by studying distribution of putative minireplicons they contained. We analyzed the 3340 bacterial plasmids for which genome sequences are available. Of the 1285 plasmids with putative known minireplicons (Additional file 4: Table S3), 34% (443 plasmids) have two or more of them (Figure 5A), indicating that plasmids fusion events are general among these plasmids. Of these 443 plasmids, 78% (345 plasmids) and 17% (75 plasmids) have 2 and 3 minireplicons, respectively. This indicates that plasmids fusion events frequently happened between two or three plasmids but rarely occur between more than three plasmids. Moreover, we compared the genome sizes between plasmids with two or more minireplicons and those with only one. Plasmids with two or more minireplicons are significant larger than plasmids with only one minireplicon (Figure 5B, P = 1.4e^-7^, Mann–Whitney test). This indicates that integrating different plasmids into a single plasmid to form larger plasmids is general during the evolution of plasmids.

In order to reduce the effect of data bias on the results, we used a subset of the plasmid sequence data to repeat the analysis. For each species that has plasmid genome sequences reported, we chose all plasmids from one strain whose plasmid number is the largest in that species. Analysis of this subset of the plasmid genome sequences confirmed the results obtained with the entire data set. Among the 771 plasmids with putative minireplicons (Additional file 5: Table S4), one third of the plasmids have two or more minireplicons (Figure 5C) and plasmids with two or more minireplicons are larger than those with only one minireplicon (Figure 5D, P = 1.035e^-06^, Mann–Whitney test).

## Conclusions

We found that megaplasmids in the *B. cereus* group larger than 100 kb contain two or more minireplicons. Minireplicons on the same plasmid usually have distinct evolutionary histories. We hypothesize that these megaplasmids are fusions of smaller plasmids. About one third of the plasmids out of the *B. cereus* group have multiple minireplicons. This indicates that plasmids fusion events occur generally during the plasmids evolutionary histories and plasmids fusion may be an important mechanism for the formation of megaplasmids.

## Methods

### Sequence collection

The genome sequences of 45 plasmids of the *B. cereus* group were retrieved from GenBank (http://www.ncbi.nlm.nih.gov) and those of 11 unpublished plasmids sequenced by our group were used in the analyses. The genome sizes of these 56 plasmids ranged from ≈20 kb to ≈566 kb (Additional file 1: Table S1). All of these data (Dataset 1) were obtained by October 20, 2012.

To study the minireplicons across all of the prokaryotic species, we collected all 3340 plasmid genome sequences from Genbank ftp site (ftp://ftp.ncbi.nlm.nih.gov/genomes/Plasmids/). These data (Dataset 2) were obtained by February 10, 2013.

### Replication essential protein sequences and minireplicons prediction

TubZ protein sequences were obtained from Dataset 1 using the hmmsearch command of the hmmer version 3.0 software [30], with an e-value<0.001, and the model Tubulin/FtsZ family (PF00091) were obtained from the Pfam database [31]. Other types of replicated protein sequences were obtained by BLASTP analysis [32] using various types of reported replication protein sequences from *B. cereus* group plasmids as query sequences and the non-redundant protein sequences from Dataset 1 as the database. A minireplicon was approved when all of the essential elements, including one or two genes encoding replication essential proteins and the DNA fragment containing origin of replication, were predicted.

We looked for replicated protein sequences from Dataset 2 by two methods; one was searching the keywords such as “replication protein”, “Rep protein” or “Primase” from the annotation files, and the other one was using hmmsearch command of hmmer software with the models associated with plasmid replication (Additional file 6: Table S2) which were downloaded from Pfam database [31]. Then we combined the results from both of the above methods, and checked these results based on public information. Minireplicons were approved when all the essential elements were predicted. All of the 1285 plasmids with putative minireplicon were showed in Additional file 4: Table S3.

### Sequence alignment and phylogenetic analysis

Protein sequences for different minireplicon of *B. cereus* group were aligned using Muscle [33]. The most disordered regions were eliminated using G-blocks [34]. The evolutionary models that best fit these sequences were determined by ProtTest version 3.0 [35], and Maximum Likelihood (ML) phylogenetic trees were generated by PhyML software version 3.0 [36], using the best fitted models (JTT + G + F for pXO1-14 and pXO1-16, LG + G + F for TubZ). Bootstrap supports were calculated as a percent of 1000 replicates. As the identity levels of TubR protein sequences among each type are very high, we collected the DNA sequences from them. Each type of *tubR* DNA sequences was aligned by Muscle and a ML tree was constructed using PhyML based on the model determined by ModelTest [37]. All the phylogenetic trees were deposited in treeBASE [38].

All statistical analyses were carried out using in-house Perl scripts and R 2.15.1 [39].

## Competing interests

The authors declare no financial or non-financial competing interests.

## Authors’ contributions

SM and ZJS designed the study with help from PDH and RLF; ZJS performed the analysis; ZJS and SM wrote the manuscript. All authors approved the final version of the manuscript.

## Supplementary Material

Additional file 1: Table S1 Plasmids analyzed in this study. Click here for file

Additional file 2 Supplementary methods and results. Click here for file

Additional file 3: Figure S4 Alignments of putative origins of replication of the four TubZ/TubR-like minireplicons. Click here for file

Additional file 4: Table S3 Plasmid and replication-associated protein information of the 1285 plasmids with putative minireplicon. Click here for file

Additional file 5: Table S4 Plasmid and replication-associated protein information of the 771 plasmids selected by host specific with putative minireplicon. Click here for file

Additional file 6: Table S2 Models associated with plasmid replication used in this study. Click here for file
