# Supplementary material for: Evolution and dynamics of megaplasmids with genome sizes larger than 100 kb in the Bacillus cereus group
Source: BMC Evol Biol. 2013 Dec 2;13:262. doi: 10.1186/1471-2148-13-262 (PMC4219350; doi:10.1186/1471-2148-13-262)
Supplement: Additional file 1: Table S1 — Plasmids analyzed in this study. [file 1471-2148-13-262-S1.doc]

**Table S1** Plasmids analyzed in this study

| plasmid | Host strain | GC% | Gene | Size (bp) | Accession number | Reference |
| --- | --- | --- | --- | --- | --- | --- |
| pXO1 | *Ba* Ames Ancestor | 32.5 | 201 | 181,677 | NC_007322 |  |
| pXO2 |  | 33 | 109 | 94,830 | NC_007323 |  |
| p03BB102_179 | *Bc* 03BB102 | 32.2 | 209 | 179,680 | NC_012473 |  |
| p03BB108_42 | *Bc 03BB108* | 36.1 | 70 | 42,470 | NZ_ABDM02000065 |  |
| p03BB108_86 |  | 32.5 | 106 | 85,879 | NZ_ABDM02000064 |  |
| p03BB108_239 |  | 31.9 | 227 | 238,933 | NZ_ABDM02000063 |  |
| p03BB108_282 |  | 30.8 | 333 | 282,009 | NZ_ABDM02000062 |  |
| pAH1134_566 | *Bc* AH1134 | 32.3 | 712 | 565,964 | NZ_ABDA02000035  NZ_ABDA02000036 |  |
| pAH187_45 | *Bc* AH187 | 35.5 | 60 | 45,173 | NC_011656 |  |
| pAH187_270 |  | 34.2 | 277 | 270,082 | NC_011655 |  |
| pPER272 | *Bc* AH818 | 33.6 | 271 | 272,145 | DQ889678 |  |
| pAH820_272 | *Bc* AH820 | 33.6 | 321 | 272,145 | NC_011777 |  |
| pBc10987 | *Bc* ATCC 10987 | 33.4 | 242 | 208,369 | NC_005707 |  |
| pBC210 | *Bc* G9241 | 31.7 | 201 | 209,385 | NC_010933 |  |
| pBCXO1 |  | 32.6 | 186 | 190,861 | NC_010934 |  |
| pCI-XO1 | *Bc* CI | 32.5 | 214 | 181,907 | NC_014331 |  |
| pCI-XO2 |  | 33.1 | 110 | 94,469 | NC_014332 |  |
| pE33L54 | *Bc* E33L | 31.9 | 57 | 53,501 | NC_007105 |  |
| pE33L466 |  | 33.1 | 447 | 466,370 | NC_007103 |  |
| pG9842_140 | *Bc* G9842 | 32.9 | 120 | 140,001 | NC_011774 |  |
| pG9842_209 |  | 30 | 251 | 209,488 | NC_011775 |  |
| pBc239 | *Bc* Q1 | 33.5 | 228 | 239,246 | NC_011973 |  |
| pBc53 |  | 35.1 | 69 | 52,766 | NC_011971 |  |
| pH308197_29 | *Bc* H3081.97 | 32.3 | 40 | 29,189 | NC_011342 |  |
| pH308197_73 |  | 30.2 | 101 | 72,792 | NC_011337 |  |
| pH308197_258 |  | 34.1 | 257 | 258,484 | NC_011339 |  |
| pLVP1401 | *Bc* VPC1401 | 31.1 | 65 | 56,149 | NC_014757 |  |
| pBMB228 | *Bt* 4AJ1 | 33 | 247 | 228,003 | - |  |
| pBtoxis | *Bt* 4Q5 | 32.4 | 124 | 127,923 | NC_010076 |  |
| pBT9727 | *Bt* 97-27 | 32.6 | 80 | 77,112 | NC_006578 |  |
| pALH1 | *Bt* Al Hakam | 36.2 | 62 | 55,939 | NC_008598 |  |
| pBMB171 | *Bt* BMB171 | 33.3 | 276 | 312,963 | NC_014172 |  |
| pCT51 | *Bt* CT-43 | 35 | 72 | 51,488 | CP001911 |  |
| pCT72 |  | 32 | 86 | 72,074 | CP001913 |  |
| pCT83 |  | 33.2 | 98 | 83,590 | CP001915 |  |
| pCT127 |  | 32.1 | 141 | 127,885 | CP001908 |  |
| pCT281 |  | 33 | 279 | 281,231 | CP001910 |  |
| pBMB46 | *Bt* HD1 | 35.4 | 67 | 46,634 | PRJNA181182^a^ |  |
| pBMB64 |  | 31.9 | 75 | 64,522 | PRJNA181182 |  |
| pBMB65 |  | 34.8 | 62 | 65,873 | PRJNA181182 |  |
| pBMB74 |  | 33.7 | 82 | 74,480 | PRJNA181182 |  |
| pBMB95 |  | 31.5 | 102 | 95,983 | PRJNA181182 |  |
| pBMB302 |  | 33.3 | 314 | 302,255 | PRJNA181182 |  |
| pBMB431 |  | 32.7 | 380 | 431,971 | PRJNA181182 |  |
| pAW63 | *Bt* HD73 | 33.8 | 75 | 71,777 | NC_010599 |  |
| pFR55 | *Bt* INTA-FR7-4 | 33.7 | 69 | 55,712 | NC_010283 |  |

**Table S1** continued.

| plasmid | Host strain | GC% | Gene | Size (bp) | Accession number | Reference |
| --- | --- | --- | --- | --- | --- | --- |
| pBMB26 | *Bt* YBT-020 | 33.1 | 192 | 187,880 | CP002509 |  |
| pBMB28 |  | 33.9 | 147 | 139,013 | CP002510 |  |
| pBMB67 | *Bt* YBT-1520 | 32.4 | 73 | 67,159 | NC_009841 |  |
| pBMB137 |  | 32.5 | 156 | 137,573 | PRJNA181183^b^ |  |
| pBMB293 |  | 33.2 | 299 | 293,574 | PRJNA181183 |  |
| pBMB400 |  | 32.5 | 385 | 416,210 | PRJNA181183 |  |
| pBWB401 | *Bw* KBAB4 | 33.7 | 332 | 417,054 | NC_010180 |  |
| pBWB402 |  | 33.3 | 75 | 75,107 | NC_010181 |  |
| pBWB403 |  | 43.4 | 76 | 64,977 | NC_010182 |  |
| pBWB404 |  | 35.4 | 71 | 52,830 | NC_010183 |  |

^a^, ^b^: As the submission of these sequences are proceeding, the bioproject IDs are used in this table. *Ba*: *Bacillus anthracis*, *Bc*: *Bacillus cereus*, *Bt*: *Bacillus thuringiensis*, *Bw*: *Bacillus weihenstephanensis*

**Supplementary References**

1. Ravel J, Jiang L, Stanley ST, Wilson MR, Decker RS, Read TD, Worsham P, Keim PS, Salzberg SL, Fraser-Liggett CM *et al*: **The complete genome sequence of *Bacillus antracis* Ames "Ancestor"**. *J Bacteriol* 2009, **191**(1):445-446.

2. Rasko DA, Rosovitz MJ, Okstad OA, Fouts DE, Jiang L, Cer RZ, Kolsto AB, Gill SR, Ravel J: **Complete sequence analysis of novel plasmids from emetic and periodontal *Bacillus cereus* isolates reveals a common evolutionary history among the B. cereus-group plasmids, including *Bacillus antracis* pXO1**. *J Bacteriol* 2007, **189**(1):52-64.

3. Rasko DA, Ravel J, Okstad OA, Helgason E, Cer RZ, Jiang L, Shores KA, Fouts DE, Tourasse NJ, Angiuoli SV *et al*: **The genome sequence of *Bacillus cereus* ATCC 10987 reveals metabolic adaptations and a large plasmid related to *Bacillus antracis* pXO1**. *Nucleic Acids Res* 2004, **32**(3):977-988.

4. Klee SR, Brzuszkiewicz EB, Nattermann H, Bruggemann H, Dupke S, Wollherr A, Franz T, Pauli G, Appel B, Liebl W *et al*: **The genome of a Bacillus isolate causing anthrax in chimpanzees combines chromosomal properties of *B. cereus* with *B. anthracis* virulence plasmids**. *PLoS One* 2010, **5**(7):e10986.

5. Han CS, Xie G, Challacombe JF, Altherr MR, Bhotika SS, Brown N, Bruce D, Campbell CS, Campbell ML, Chen J *et al*: **Pathogenomic sequence analysis of *Bacillus cereus* and *Bacillus thuringiensis* isolates closely related to *Bacillus antracis***. *J Bacteriol* 2006, **188**(9):3382-3390.

6. Xiong Z, Jiang Y, Qi D, Lu H, Yang F, Yang J, Chen L, Sun L, Xu X, Xue Y *et al*: **Complete genome sequence of the extremophilic *Bacillus cereus* strain Q1 with industrial applications**. *J Bacteriol* 2009, **191**(3):1120-1121.

7. Berry C, O'Neil S, Ben-Dov E, Jones AF, Murphy L, Quail MA, Holden MT, Harris D, Zaritsky A, Parkhill J: **Complete sequence and organization of pBtoxis, the toxin-coding plasmid of *Bacillus thuringiensis* subsp. *israelensis***. *Appl Environ Microbiol* 2002, **68**(10):5082-5095.

8. Challacombe JF, Altherr MR, Xie G, Bhotika SS, Brown N, Bruce D, Campbell CS, Campbell ML, Chen J, Chertkov O *et al*: **The complete genome sequence of *Bacillus thuringiensis* Al Hakam**. *J Bacteriol* 2007, **189**(9):3680-3681.

9. He J, Shao X, Zheng H, Li M, Wang J, Zhang Q, Li L, Liu Z, Sun M, Wang S *et al*: **Complete genome sequence of *Bacillus thuringiensis* mutant strain BMB171**. *J Bacteriol* 2010, **192**(15):4074-4075.

10. He J, Wang J, Yin W, Shao X, Zheng H, Li M, Zhao Y, Sun M, Wang S, Yu Z: **Complete genome sequence of *Bacillus thuringiensis* subsp. *chinensis* strain CT-43**. *J Bacteriol* 2011, **193**(13):3407-3408.

11. Van der Auwera GA, Andrup L, Mahillon J: **Conjugative plasmid pAW63 brings new insights into the genesis of the *Bacillus antracis* virulence plasmid pXO2 and of the *Bacillus thuringiensis* plasmid pBT9727**. *BMC Genomics* 2005, **6**:103.

12. Amadio AF, Benintende GB, Zandomeni RO: **Complete sequence of three plasmids from *Bacillus thuringiensis* INTA-FR7-4 environmental isolate and comparison with related plasmids from the *Bacillus cereus* group**. *Plasmid* 2009, **62**(3):172-182.

13. Zhu Y, Shang H, Zhu Q, Ji F, Wang P, Fu J, Deng Y, Xu C, Ye W, Zheng J *et al*: **Complete genome sequence of *Bacillus thuringiensis* serovar *finitimus* strain YBT-020**. *J Bacteriol* 2011, **193**(9):2379-2380.

14. Chao L, Qiyu B, Fuping S, Ming S, Dafang H, Guiming L, Ziniu Y: **Complete nucleotide sequence of pBMB67, a 67-kb plasmid from *Bacillus thuringiensis* strain YBT-1520**. *Plasmid* 2007, **57**(1):44-54.
