# Supplementary material for: Evolution and dynamics of megaplasmids with genome sizes larger than 100 kb in the Bacillus cereus group
Source: BMC Evol Biol. 2013 Dec 2;13:262. doi: 10.1186/1471-2148-13-262 (PMC4219350; doi:10.1186/1471-2148-13-262)
Supplement: Additional file 2 — Supplementary methods and results. [file 1471-2148-13-262-S2.doc]

**Supplementary methods**

**Cloning and validation of two novel TubZ/TubR minireplicons**

Luria-Bertani (LB) broth was used to culture *Escherichia coli* DH5α at 37°C, 200 rev min^−1^, and *Bacillus thuringiensis* strains at 28°C, 200 rev min^−1^. Plasmid DNA purified from *E. coli*, transformation of *E. coli*, and recombinant DNA techniques were carried out using standard methods . Plasmid DNA from *B. thuringiensis* was extracted as described by Reyes-Ramirez and Ibarra .

Two DNA fragments that were predicted to contain the two novel TubZ/TubR minireplicons were obtained from *B. thuringiensis* plasmids pBMB28 and pBMB228 by PCR using primers p28F/p28R (5′–AACATGGTCGTATCAAGGAT-3′ / 5′- TAGGTCGCCATCACAATAC-3′) and p228F/p228R (5′- ATGTCTGTCCATAGTGTCAT-3′ / 5′- CCGCCGCAATAGTTATATG-3′), respectively. For each amplification reaction mixture, 10 ng of plasmid DNA was subjected to PCR in a total volume of 25 µL, with 2.5 U Ex Taq polymerase (Takara Bio, Otsu, Japan), 2.5 µL 10 × PCR buffer, 1.5 µL dNTP mixture (25 mM), and 0.8 µM of each primer. The conditions for PCR amplification were as follows: 95°C for 5 minutes; 30 cycles of 95°C for 30 s, 50°C (pBMB28) or 49°C (pBMB228) for 30 s, 72°C for 3 minutes; 72°C for 10 minutes. PCR products were purified using a Gel Extraction Kit (Omega Bio-Tek, Norcross，Georgia, USA). Amplicons were cloned into pMD 18-T Vector (Takara Bio) by TA cloning technique to generate plasmids pEMB28R and pEMB228R, and then transformed into *E. coli* DH5α. The two constructed plasmids were then extracted from *E. coli*, digested with restriction endonucleases *Sac*I and *Pst*I, and cloned into *E. coli* vector pDG780 , containing ColE1 and ampicillin and kanamycin resistance genes, to generate plasmids pBMB28R and pBMB228R (Figure S1). The two plasmids were electro-transformed into *B. thuringiensis* strain BMB171 using the protocol described by Peng *et al*. . Transformants were cultured in LB medium with a kanamycin concentration of 50 µg/mL at 28°C to mid-log phase. Plasmids were extracted from these transformants and used as templates for PCR using primer pairs p28-TubZ-1 / p28-TubZ-2 (5′-TCAAGCAGAAGAGAGACGA-3′ / 5′-AATAGGCACACCTGGTGTGA-3′) and p228-TubZ-1/p228-TubZ-2 (5′-TAGGTGGAGGTACTGGAAC-3′ / 5′-AATGTCTTCTTCGGCTCTG-3′), respectively. For each amplification reaction mixture, 10 ng of plasmid DNA was subjected to PCR in a total volume of 20 µL, with 2 U Taq polymerase (Takara Bio), 2 µL 10 × PCR buffer, 0.5 µL dNTP mixture (25 mM), and 0.8 µM of each primer. The conditions for both the above PCR amplifications were as follows: 95°C for 5 minutes; 30 cycles of 95°C for 30 s, 50°C for 30 s, 72°C for 1 minute; 72°C for 5 minutes. Plasmids pBMB28 and pBMB228 were used as positive controls. Successful transformation of the correct amplicon was validated by comparison to the positive control (Figure S2).

To determinate the minimum region for plasmid replication, a point mutation of the initiation codon that led to premature termination of translation for each gene in the two minireplicons was constructed using splicing by overhang extension PCR (SOE PCR) and verified by sequencing. The SOE PCR was carried out using standard methods . For mutation of *tubR* in Rep466, four primers were used: p28F, p28R, p28MR-1 (5′-AATACCTCGTTCtAGTTGTC-3′), and p28MR-2 (5′-GACAACTaGAACGAGGTATT-3′), and the start codon were transferred from TTG to stop codon TAG. For mutation of *tubZ* in Rep466, along with p28F and p28R, primers p28MZ-1 (5′-TGCCtaTATTTTATCTCTCCCT-3′) and p28MZ-2 (5′-AGGGAGAGATAAAATAtaGGCA-3′) were used to replace the start codon with the stop codon TAG. For the two genes in Rep228, mutation of *tubR* included four primers, p228F, p228R, p228MR-1 (5′-AAGCTTCATCCtaTCCTATC-3′) and p228MR-2 (5′-GATAGGAtaGGATGAAGCTT-3′), and its start codon, ATG, was replaced by a stop codon, TAG. For the mutation of the other gene, *tubZ*, in Rep228, there were also four primers: p228F, p228R, p228MZ-1 (5′-CAAACtaTATATTTCCTCCTC-3′) and p228MZ-2 (5′-GAGGAGGAAATATAtaGTTTG-3′). The replication functions of these plasmids were tested like those for pBMB28R and pBMB228R.

**Supplementary results**

**Two novel TubZ/TubR minireplicons were confirmed on different megaplasmids of the *B. cereus* group, respectively**

To date, two TubZ/TubR minireplicons have been reported on plasmids from the *B. cereus* group. One was located on pBtoxis, which contained two replication proteins, ORF156 and ORF157 . The other one was on pXO1, which contained one replication protein, RepX . Both ORF156 and RepX are TubZ-like proteins and contain the conserved domain, FtsZ/tubulin-like NTPase. Novel minireplicons were predicted by searching for this domain in protein sequences from plasmids of the *B. cereus* group. We predicted two new such minireplicons, one of which was designated Rep228 and consisted of a TubZ protein and a putative TubR protein with no predicted DNA binding domain, while the other was named Rep466 and contained two proteins, TubZ and a putative TubR which showed very low level similarity to a DNA binding protein.

The two putative minireplicons containing Rep228 from pBMB228 and Rep466 from pBMB28 were amplified from two different *B. thuringiensis* strains and transferred to plasmid-less strain BMB171. As shown in Figure S3, these two putative minireplicons were functional for replication of plasmids. For each minireplicon, mutation tests showed that the two genes were both essential for replication (Figures S3A and S3C). The detail methods for these verifications were described in supplementary methods. Putative replication origins were also predicted for both of the minireplicons (Figures S3B and S3D).

**Supplementary figures**

**Figure S1**


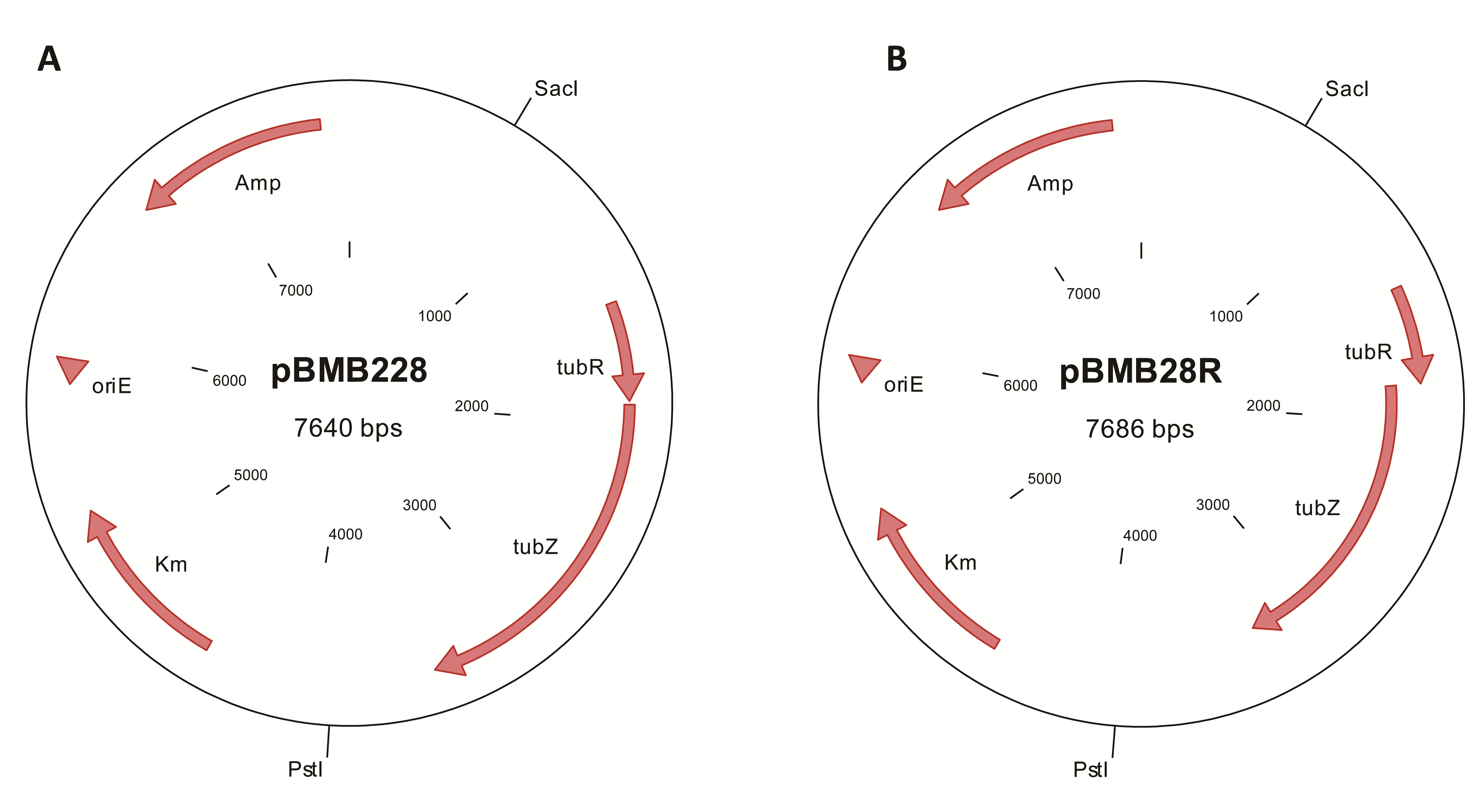


**Figure S1 Maps of the two plasmids which can replicate in *B. thuringiensis* BMB171.** (A) pBMB228R contained the minireplicon Rep228 which was cloned from the plasmid pBMB228. (B) pBMB28R contained the minireplicon Rep488 which was cloned from the plasmid pBMB28.

**Figure S2**


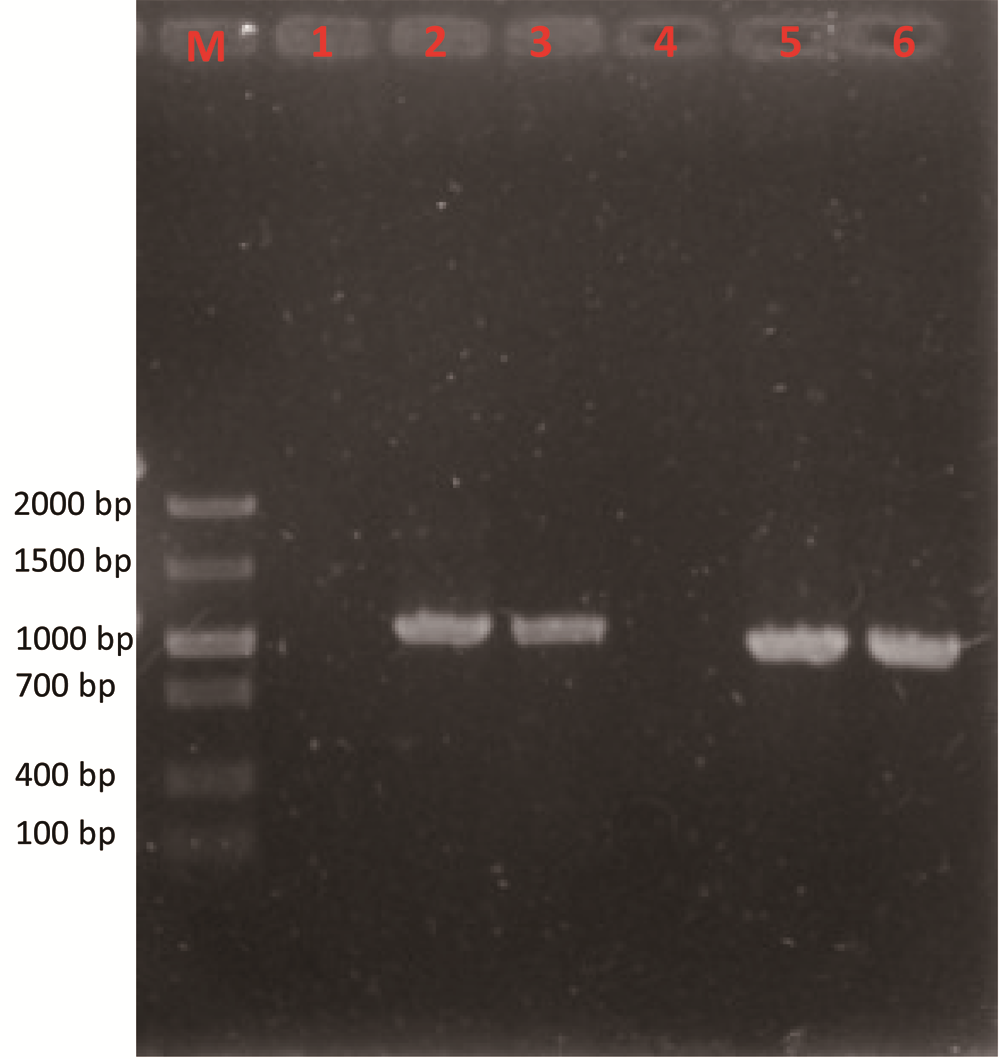


**Figure S2 PCR test of pBMB228R and pBMB28R transformants.** PCR-amplificates of isolated plasmid DNA from untransformed *B. thuringiensis* BMB171 (lane 1 and lane 4), *B. thuringiensis* BGSC 4AJ1 containing pBMB228 (lane 2), *B. thuringiensis* BMB171 containing pBMB228R (lane 3), *B. thuringiensis* YBT-020 containing pBMB28 (lane 5) and *B. thuringiensis* BMB171 containing pBMB28R 171 (lane 6). PCR products of the expected size were obtained for all transformants (1033 bp for pBMB228R and 922 bp for pBMB28R).

**Fig. S4**

**
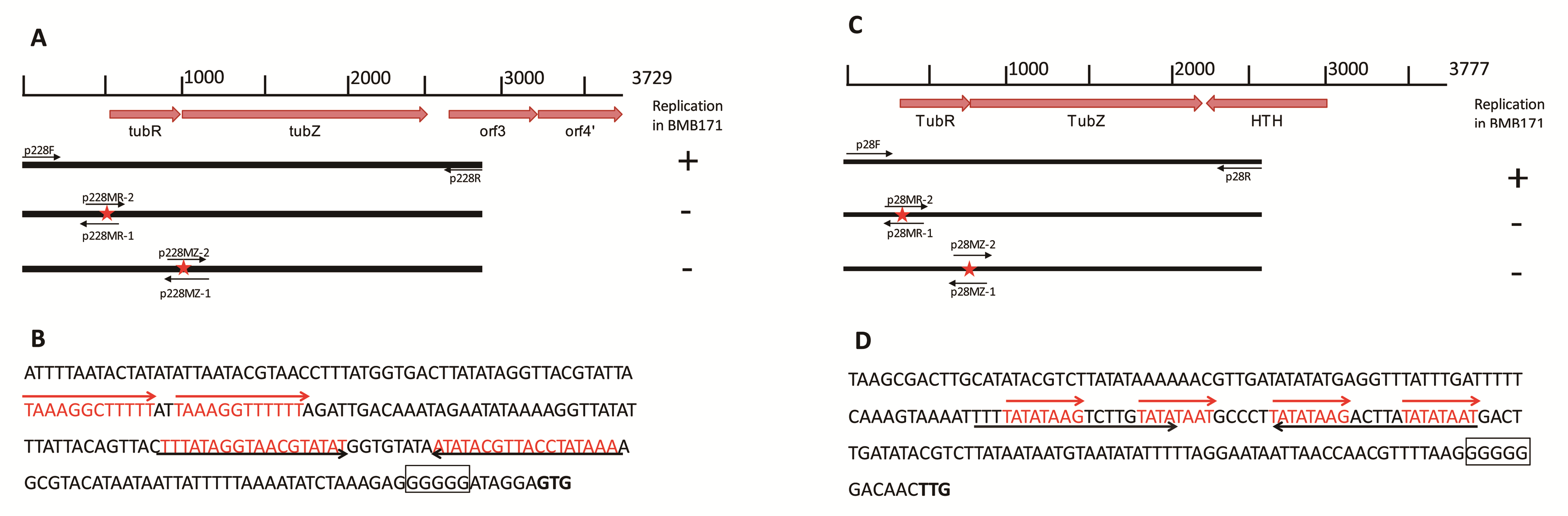
**

**Figure S3 Two novel minireplicons and their putative origins of replication from plasmids of the *B. cereus* group.** (A) The minimal region of pBMB228 (Rep228) replication consists of two genes, *tubR* and *tubZ*, and these two genes are from the same operon. Both genes are essential for replication of the recombinant plasmid in *B. thuringiensis* BMB171. (B) Putative origin of replication of Rep228 is located upstream of the two essential genes. Two 12-bp direct repeats and two 18-bp inverted repeats are found in this AT-rich region. (C) Minimal minireplicon of pBMB28 (Rep466), an operon containing two genes, *tubR* and *tubZ*, is essential for replication. (D) The putative origin of replication of rep466 in located in an AT-rich region upstream of the operon. Four 8-bp imperfect direct repeats and two 20-bp inverted repeats are overlapped in this region. Arrows in (A) and (C) indicate positions of PCR primers. Point mutations are marked by red stars in (A) and (C). In (B) and (D), direct repeats are marked by arrows in the same direction and inverted repeats by opposite arrows, ribosomal binding sites are marked by boxes, and the start codons are showed in bold.

**Supplementary References**

1. Sambrook J, Russell DW: **Molecular Cloning: A Laboratory Manual, 3rd edn.** New York: Cold Spring Harbor Laboratory Press; 2001.

2. Reyes-Ramirez A, Ibarra JE: **Plasmid patterns of *Bacillus thuringiensis* type strains**. *Appl Environ Microbiol* 2008, 74(1):125-129.

3. Huang J, Guo S, Mahillon J, Van der Auwera GA, Wang L, Han D, Yu Z, Sun M: **Molecular characterization of a DNA fragment harboring the replicon of pBMB165 from *Bacillus thuringiensis* subsp. *tenebrionis***. *BMC Genomics* 2006, 7:270.

4. Peng D, Luo Y, Guo S, Zeng H, Ju S, Yu Z, Sun M: **Elaboration of an electroporation protocol for large plasmids and wild-type strains of *Bacillus thuringiensis***. *J Appl Microbiol* 2009, 106(6):1849-1858.

5. Ho SN, Hunt HD, Horton RM, Pullen JK, Pease LR: **Site-directed mutagenesis by overlap extension using the polymerase chain reaction**. *Gene* 1989, 77(1):51-59.

6. Tang M, Bideshi DK, Park HW, Federici BA: **Minireplicon from pBtoxis of *Bacillus thuringiensis* subsp. *israelensis***. *Appl Environ Microbiol* 2006, 72(11):6948-6954.

7. Tinsley E, Khan SA: **A novel FtsZ-like protein is involved in replication of the anthrax toxin-encoding pXO1 plasmid in *Bacillus anthracis***. *J Bacteriol* 2006, 188(8):2829-2835.
