# Supplementary material for: Evolution and dynamics of megaplasmids with genome sizes larger than 100 kb in the Bacillus cereus group
Source: BMC Evol Biol. 2013 Dec 2;13:262. doi: 10.1186/1471-2148-13-262 (PMC4219350; doi:10.1186/1471-2148-13-262)
Supplement: Additional file 3: Figure S4 — Alignments of putative origins of replication of the four TubZ/TubR-like minireplicons. [file 1471-2148-13-262-S3.docx]

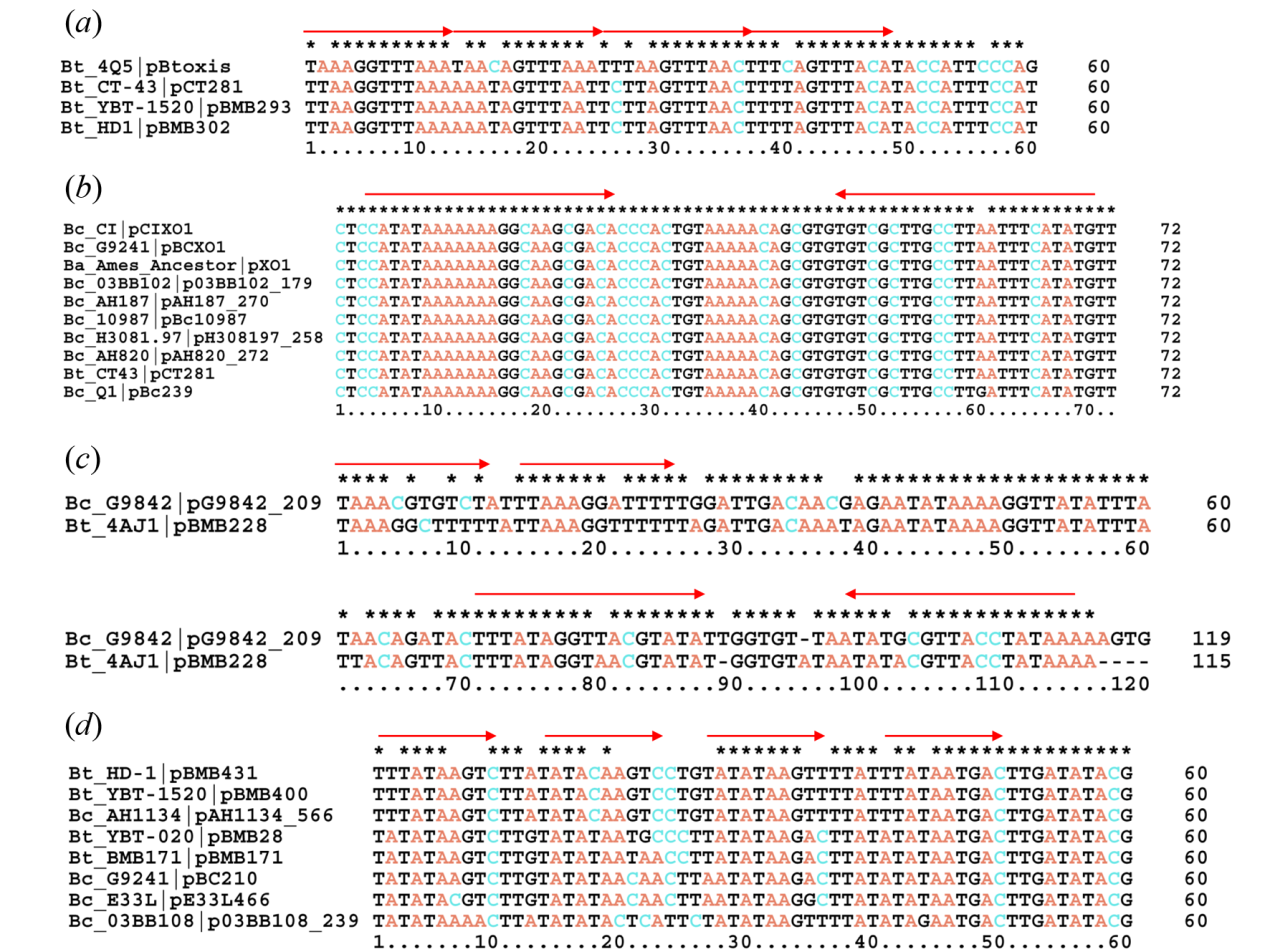


**Figure S4** Alignments of putative replication origins of the four TubZ/TubR-like minireplicons. (a) ORF156/ORF157, four 12-bp imperfect direct repeats; (b) RepX, two 23-bp inverted repeats; (c) Rep228, two 12-bp direct repeats and two 18-bp inverted repeats; (d) Rep466, four 8-bp imperfect direct repeats.
