# Supplementary material for: Evolution and dynamics of megaplasmids with genome sizes larger than 100 kb in the Bacillus cereus group
Source: BMC Evol Biol. 2013 Dec 2;13:262. doi: 10.1186/1471-2148-13-262 (PMC4219350; doi:10.1186/1471-2148-13-262)
Supplement: Additional file 6: Table S2 — Models associated with plasmid replication used in this study. [file 1471-2148-13-262-S6.docx]

**Table S2** Models associated with plasmid replication used in this study

| Accession | ID | Description |
| --- | --- | --- |
| [PF00091](http://pfam.sanger.ac.uk/family/PF00091) | [Tubulin](http://pfam.sanger.ac.uk/family/PF00091) | Tubulin/FtsZ family, GTPase domain |
| [PF00772](http://pfam.sanger.ac.uk/family/PF00772) | [DnaB](http://pfam.sanger.ac.uk/family/PF00772) | DnaB-like helicase N terminal domain |
| [PF01051](http://pfam.sanger.ac.uk/family/PF01051) | [Rep_3](http://pfam.sanger.ac.uk/family/PF01051) | Initiator Replication protein |
| [PF01446](http://pfam.sanger.ac.uk/family/PF01446) | [Rep_1](http://pfam.sanger.ac.uk/family/PF01446) | Replication protein |
| [PF01719](http://pfam.sanger.ac.uk/family/PF01719) | [Rep_2](http://pfam.sanger.ac.uk/family/PF01719) | Plasmid replication protein |
| [PF02195](http://pfam.sanger.ac.uk/family/PF02195) | [ParBc](http://pfam.sanger.ac.uk/family/PF02195) | ParB-like nuclease domain |
| [PF02387](http://pfam.sanger.ac.uk/family/PF02387) | [IncFII_repA](http://pfam.sanger.ac.uk/family/PF02387) | IncFII RepA protein family |
| [PF02486](http://pfam.sanger.ac.uk/family/PF02486) | [Rep_trans](http://pfam.sanger.ac.uk/family/PF02486) | Replication initiation factor |
| [PF03090](http://pfam.sanger.ac.uk/family/PF03090) | [Replicase](http://pfam.sanger.ac.uk/family/PF03090) | Replicase family |
| [PF03428](http://pfam.sanger.ac.uk/family/PF03428) | [RP-C](http://pfam.sanger.ac.uk/family/PF03428) | Replication protein C N-terminal domain |
| [PF03796](http://pfam.sanger.ac.uk/family/PF03796) | [DnaB_C](http://pfam.sanger.ac.uk/family/PF03796) | DnaB-like helicase C terminal domain |
| [PF04796](http://pfam.sanger.ac.uk/family/PF04796) | [RepA_C](http://pfam.sanger.ac.uk/family/PF04796) | Plasmid encoded RepA protein |
| [PF05086](http://pfam.sanger.ac.uk/family/PF05086) | [Dicty_REP](http://pfam.sanger.ac.uk/family/PF05086) | Dictyostelium (Slime Mold) REP protein |
| [PF05732](http://pfam.sanger.ac.uk/family/PF05732) | [RepL](http://pfam.sanger.ac.uk/family/PF05732) | Firmicute plasmid replication protein (RepL) |
| [PF06430](http://pfam.sanger.ac.uk/family/PF06430) | [L_lactis_RepB_C](http://pfam.sanger.ac.uk/family/PF06430) | Lactococcus lactis RepB C-terminus |
| [PF06504](http://pfam.sanger.ac.uk/family/PF06504) | [RepC](http://pfam.sanger.ac.uk/family/PF06504) | Replication protein C (RepC) |
| [PF06613](http://pfam.sanger.ac.uk/family/PF06613) | [KorB_C](http://pfam.sanger.ac.uk/family/PF06613) | KorB C-terminal beta-barrel domain |
| [PF06970](http://pfam.sanger.ac.uk/family/PF06970) | [RepA_N](http://pfam.sanger.ac.uk/family/PF06970) | Replication initiator protein A (RepA) N-terminus |
| [PF07042](http://pfam.sanger.ac.uk/family/PF07042) | [TrfA](http://pfam.sanger.ac.uk/family/PF07042) | TrfA protein |
| [PF07232](http://pfam.sanger.ac.uk/family/PF07232) | [DUF1424](http://pfam.sanger.ac.uk/family/PF07232) | Putative rep protein (DUF1424) |
| [PF07261](http://pfam.sanger.ac.uk/family/PF07261) | [DnaB_2](http://pfam.sanger.ac.uk/family/PF07261) | Replication initiation and membrane attachment |
| [PF07506](http://pfam.sanger.ac.uk/family/PF07506) | [RepB](http://pfam.sanger.ac.uk/family/PF07506) | RepB plasmid partitioning protein |
| [PF08048](http://pfam.sanger.ac.uk/family/PF08048) | [RepA1_leader](http://pfam.sanger.ac.uk/family/PF08048) | Tap RepA1 leader peptide |
| [PF08273](http://pfam.sanger.ac.uk/family/PF08273) | [Prim_Zn_Ribbon](http://pfam.sanger.ac.uk/family/PF08273) | Zinc-binding domain of primase-helicase |
| [PF08535](http://pfam.sanger.ac.uk/family/PF08535) | [KorB](http://pfam.sanger.ac.uk/family/PF08535) | KorB domain |
| [PF08707](http://pfam.sanger.ac.uk/family/PF08707) | [PriCT_2](http://pfam.sanger.ac.uk/family/PF08707) | Primase C terminal 2 (PriCT-2) |
| [PF08708](http://pfam.sanger.ac.uk/family/PF08708) | [PriCT_1](http://pfam.sanger.ac.uk/family/PF08708) | Primase C terminal 1 (PriCT-1) |
| [PF08857](http://pfam.sanger.ac.uk/family/PF08857) | [ParBc_2](http://pfam.sanger.ac.uk/family/PF08857) | Putative ParB-like nuclease |
| [PF10134](http://pfam.sanger.ac.uk/family/PF10134) | [RPA](http://pfam.sanger.ac.uk/family/PF10134) | Replication initiator protein A |
| [PF10609](http://pfam.sanger.ac.uk/family/PF10609) | [ParA](http://pfam.sanger.ac.uk/family/PF10609) | ParA/MinD ATPase like |
| [PF11740](http://pfam.sanger.ac.uk/family/PF11740) | [KfrA_N](http://pfam.sanger.ac.uk/family/PF11740) | Plasmid replication region DNA-binding N-term |
| [PF11800](http://pfam.sanger.ac.uk/family/PF11800) | [RP-C_C](http://pfam.sanger.ac.uk/family/PF11800) | Replication protein C C-terminal region |
| [PF13010](http://pfam.sanger.ac.uk/family/PF13010) | [pRN1_helical](http://pfam.sanger.ac.uk/family/PF13010) | Primase helical domain |
| [PF13814](http://pfam.sanger.ac.uk/family/PF13814) | [Replic_Relax](http://pfam.sanger.ac.uk/family/PF13814) | Replication-relaxation |
